# DSAIL power management board: Powering the Raspberry Pi autonomously off the grid

**DOI:** 10.1016/j.ohx.2022.e00337

**Published:** 2022-07-13

**Authors:** Gabriel Kiarie, Jason Kabi, Ciira wa Maina

**Affiliations:** Centre for Data Science and Artificial Intelligence (DSAIL), Dedan Kimathi University of Technology, Nyeri, Kenya

**Keywords:** Sensor power management, Solar power, Ecological data collection, Long term deployment

## Abstract

The Raspberry Pi is a credit card sized single board computer that finds its use in very diverse projects. Being a computer it runs on a full operating system and can be interfaced with a wide range of hardware. Its ability to collect and store data and its superior processing capabilities gives it an edge over other microprocessors. When used to collect data away from the grid, alternative methods of powering the Raspberry Pi have to be used. An ideal powering system should be autonomous, allowing the Raspberry Pi to be deployed indefinitely without the need to check on the system due to power shortcomings. In this paper we introduce the DSAIL Power Management Board that is used to power the Raspberry Pi autonomously. We have developed a prototype and used it to collect ecological data from a conservancy in Central Kenya.

## Graphical abstract

**Figure:**
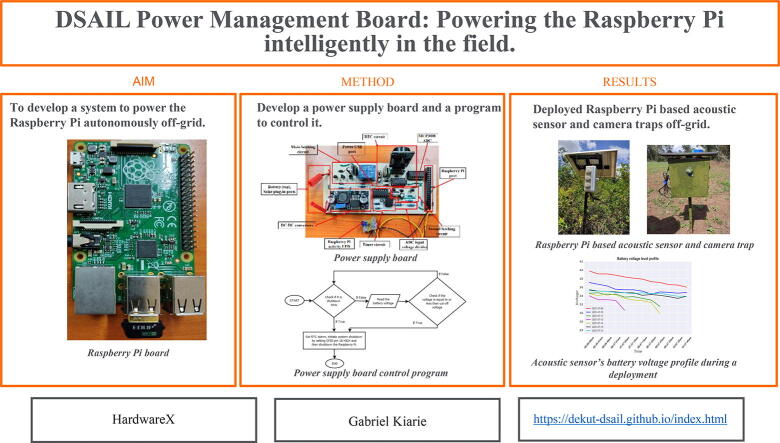

## Specifications table

**Table:** 

Hardware name	DSAIL Power Management Board
Subject area	Electrical and Electronic Engineering
Hardware type	Electronic engineering
Closest commercial analog	PiJuice
Open source licence	CC BY 4.0
Cost of hardware	KES 3000
Source file repository	https://data.mendeley.com/datasets/pr2d4yn4dd/2

## Hardware in context

Machine learning is gaining wide usage in many walks of life. The adoption of data-intensive machine learning algorithms is seeing new applications without enough labeled data. Data collection is one of the main challenges in machine learning [1]. Occasionally, these data need to be collected from the environments far from the grid using sensors. To power these sensors, we have to resort to alternative energy sources such as batteries, solar or wind energy. For reliable powering of the sensors, batteries are the most effective due to the intermittent nature of the other alternatives stated above [2]. One of the main challenges encountered when designing battery-powered sensors to be deployed in the field is lengthening their duration of operation. The batteries can be coupled with the other sources of energy such as solar and wind to allow long term deployment and smooth operation of the sensors [3].

The Raspberry Pi is a single board computer that is operated using a Unix-based operating system that is freely distributed and can be powered from DC sources like batteries [4], [5]. It is an ideal device for data collection since it can be interfaced with a lot of sensors and it also has the ability to process the data onboard. The Raspberry Pi being a computer means care needs to be taken when powering it [6]. Shutting it down unconventionally due to depletion of batteries may result in corruption of its storage hence loss of data.

The existing technology of powering the Raspberry Pi away from the grid includes: (1) using a high output power bank; (2) and use of a power management board like the PiJuice UPS HAT. These methods, however, are not suitable to power the Raspberry Pi for long periods of time in the field. The DSAIL Power Management Board is designed to power the Raspberry Pi autonomously for long periods therefore allowing long-term data collection deployments off the grid. This paper fully describes the board.

## Hardware description

The DSAIL Power Management Board is a 14.2 cm by 6.3 cm printed circuit board (PCB). The main components on the PCB are DC converters, a mechanical relay, a DS3231 real time clock (RTC), a timer circuit, an analog-to-digital converter (ADC) and MOSFET latching circuits. The system is designed to power the Raspberry Pi using the common 3.7 V lithium polymer (LiPo) battery and a solar panel. The idea was to design an autonomous power supply board that should be able to power the Raspberry Pi taking into consideration the intermittent nature of solar energy as a source of power.

The board enables safe shutdown of the Raspberry Pi when the battery used to power it gets drained and wakes up when the battery gets charged. To achieve this, the board has an ADC that enables the Raspberry Pi to monitor the voltage level hence the charge of the battery. Before the system shuts down, it schedules to wake up later after the batteries have been charged by setting an alarm of the RTC. The RTC plays the role of waking the system and also setting the time of the Raspberry Pi on boot.

It is common to also find some cases where we need to deploy the Raspberry Pi to collect data during a specific time of the day. Using our power management board, it is possible to set the system to wake up and operate during the required period only. Once the period is over, the system will shut down and only wake up the following day at the required time. One can also create multiple windows of operation within a single day in between which the system is shut down. We have successfully deployed a camera trap based on this system with two windows of operation for image data collection. This makes the system work optimally by preventing wastage of power when the system is not collecting data. This autonomy of the system in the field cannot be achieved when using a power bank or the PiJuice to power the Raspberry Pi.

## Design files

The design files consist of all the files needed to make and run the DSAIL Power Management Board. Table 1 shows the list of design files.

**Table 1:** Design files.

Design file name	File type	Open source licence	Location of the file
Hardware	PCB designs	CC BY 4.0	https://data.mendeley.com/datasets/pr2d4yn4dd/2
Software	Programs	CC BY 4.0	https://data.mendeley.com/datasets/pr2d4yn4dd/2

### Hardware

The Hardware folder contains the KiCad EDA design files. The **hat2.pro** is the KiCad project file for the hardware design. To open the file, download and install KiCad EDA software (https://www.kicad.org/download/). After installing KiCad open the hat2.pro file and select to view the schematic or the PCB design of the hardware.

### Software

The software comprises instructions of how to prepare the Raspberry Pi and programs to enable it to work with the power supply board. All software can be accessed from the hardware GitHub repository (https://github.com/DeKUT-DSAIL/powering-raspberrypi).

Bill of materials summary.

Table 2 shows the components that are needed to reproduce the board.

**Table 2:** Bill of materials.

**Designator**	**Component**	**Number**	**Cost per unit (KES)**	**Total cost (KES)**	**Source of materials**	**Material type**
J3, J5, J9	JST Connector	3	5	15	Nerokas	Electrical
U3	LM2596	1	200	200	Pixel Electric	Electronic
U4	MT3608	1	100	100	Pixel Electric	Electronic
K1	SRD-05VDC-SL-C	1	50	50	Pixel Electric	Electronic
D4, D1, D2, D3	Diode (IN4001)	4	5	20	Pixel Electric	Electronic
Q5, Q1	IRF540	2	100	200	Nerokas	Electronic
C2, C5, C1	200μF capacitor	2	10	20	Pixel Electric	Electronic
C1	4.7μF capacitor	1	5	5	Pixel Electric	Electronic
C3	10μF capacitor	1	5	5	Pixel Electric	Electronic
C4	100 nF capacitor	1	5	5	Pixel Electric	Electronic
Q6, Q7, Q2, Q4	PN2222A	4	5	20	Pixel Electric	Electronic
Q8, Q3	2N3906	2	5	10	Pixel Electric	Electronic
J2	Conn_01x02	2	2	4	Pixel Electric	Electrical
R10, R4	1 kΩ	2	2	4	Pixel Electric	Electrical
R5, R11	3.3 kΩ	3	2	6	Pixel Electric	Electrical
R8, R9, R3	470 Ω	2	2	4	Pixel Electric	Electrical
R1, R2	1 MΩ	2	2	4	Pixel Electric	Electrical
R6, R7	20 kΩ	1	5	5	Pixel Electric	Electrical
S1	Toggle-slide switch	1	50	50	Pixel Electric	Electrical
U1	LD1117AV33	1	350	350	Pixel Electric	Electronic
U2	MCP3008	1	300	300	Pixel Electric	Electronic
U3	DS3231	1	50	50	Pixel Electric	Electronic
U4	NE555 IC	1	30	30	Pixel Electric	Electronic
U5	SN74HC04N	1	30	30	Pixel Electric	Electronic
U6	CD4017BE	1	80	80	Pixel Electric	Electronic
	3.7V, 6600mAh LiPo	1	1400	1400	Pixel Electric	Electrical
	LED	1	5	5	Pixel Electric	Electronic

## Build instructions

The following are the function blocks that make the DSAIL Power Management Board:

### Switches

**Fig. 1:**
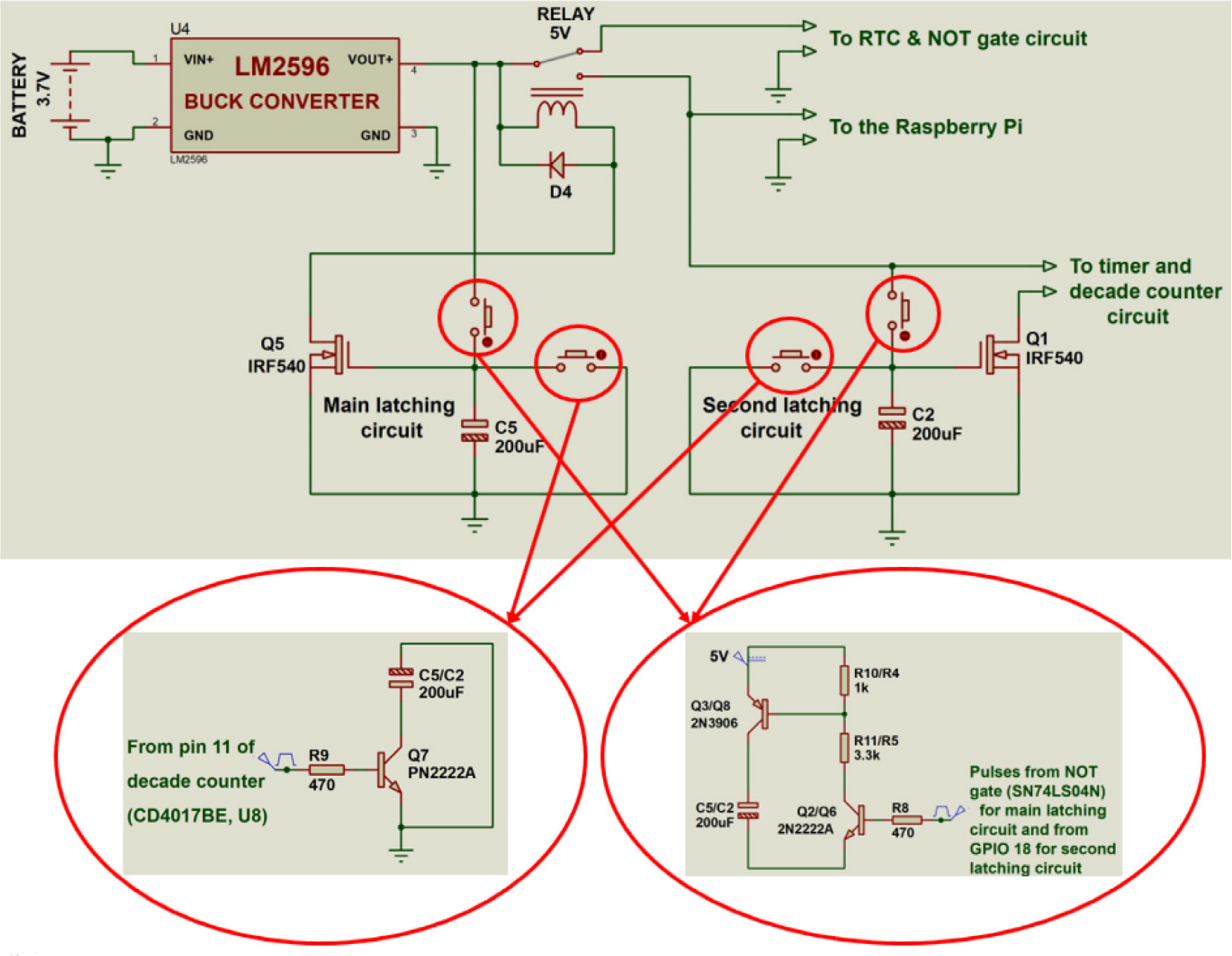
MOSFET based latching circuits responsible for switching the entire board and the timer circuit ON or OFF. The latching circuits are controlled using BJT switches.

The switches are based on IRF540 *N*-channel MOSFETS latching circuits. They are responsible for switching different sections of the circuit or the entire system ON or OFF. Special BJT switches are used to control the MOSFET latches making it possible to drive the circuit using electrical pulses from the Raspberry Pi, the DS3231 alarm and a decade counter. This property greatly enhances the autonomy property of the board. The board has two latching circuits: the main latching circuit responsible for switching the whole board ON or OFF and the second latching circuit that switches the timer section ON and OFF. Fig. 1 shows the latching circuits of the system.

### Battery monitoring circuit

This section has the MCP3008 ADC and a voltage divider. The ADC enables the Raspberry Pi to continuously read the voltage of the battery. The Raspberry Pi lacks an onboard ADC hence the need for an external ADC to read analog inputs like the voltage of a battery. Batteries have rated maximum depth of discharge (MDOD) above which they should not be discharged in order to lengthen their lifespan. Different types of batteries have different MDOD ratings. The MDOD of lithium polymer batteries is 80 %. It is not easy to determine the depth of discharge (DOD) of a battery directly. As charge is being drawn from a battery, the nominal voltage of the battery usually drops. Using the voltage of the battery, we can approximate the depth of discharge of the battery. The cut-off voltage of a battery is the voltage beyond which the battery’s DOD exceeds its rated MDOD. Therefore, we can prevent over discharge of a battery by ensuring the voltage is always above the rated cut-off voltage [7].

**Fig. 2:**
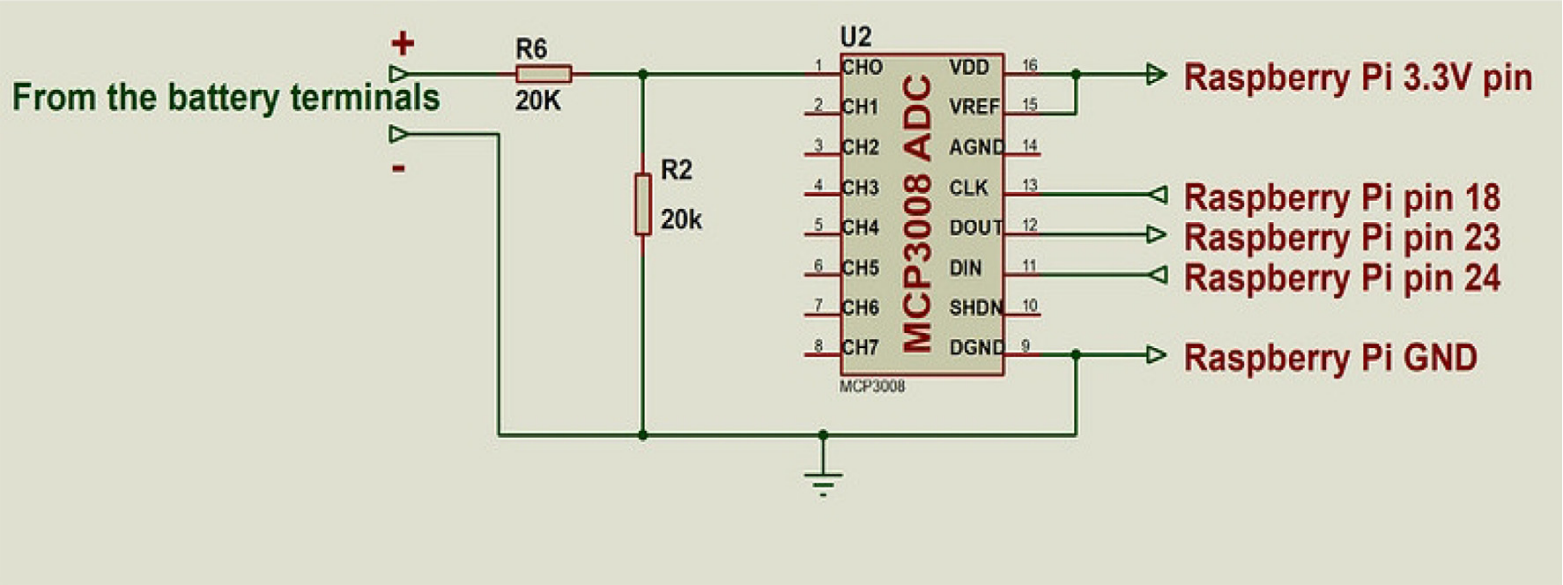
MCP3008 ADC circuit to monitor battery state.

By reading the voltage of the battery, the Raspberry Pi is able to monitor the state of the battery. When the voltage of the battery drops to the cut-off voltage, the Raspberry Pi initiates the shutdown process of the board and itself. Fig. 2 shows the battery monitoring circuit of the board.

### RTC circuit

**Fig. 3:**
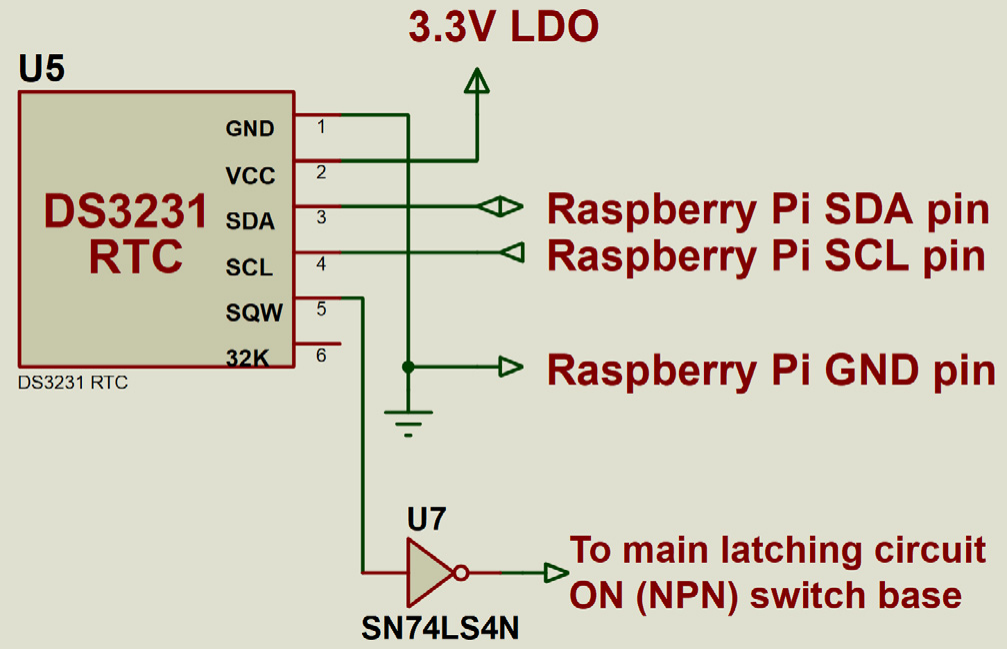
RTC circuit. A NOT gate interfaces the active low DS3231 RTC alarm interrupt pin with the main latching circuit.

The RTC circuit plays two major roles in the system. Firstly, setting the time and date on the Raspberry Pi on boot since it lacks an onboard RTC and secondly, waking the system after it has shut down. The DS3231 RTC used on this board has an alarm interrupt that we leverage for the latter. Before the Raspberry Pi shuts down, it sets an alarm on the RTC to schedule wake up. The alarm’s interrupt is used to trigger the system to wake up when the RTC time matches with the set alarm time. The alarm interrupt is active low hence a NOT gate is used to interface the RTC and the main MOSFET latching circuit. Fig. 3 shows the RTC circuit.

### Timer circuit

**Fig. 4:**
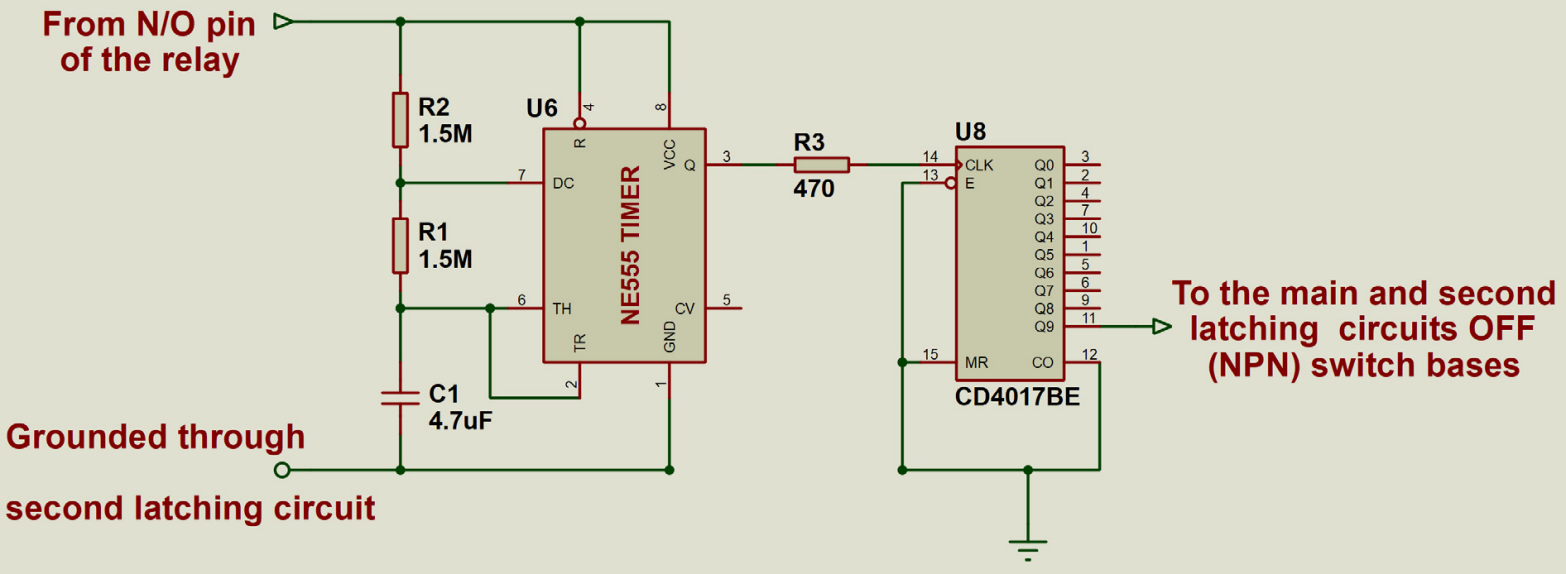
555 Timer circuit configured in astable mode. It output is connected to the decade counter that count the pulses and trigger shutting down of the board at the 10th pulse.

The timer circuit allows the Raspberry Pi to shut down safely before power is cut off. The timer circuit comprises a 555 timer and a decade counter. The timer is configured in astable mode and it produces pulses with a period of 4 s. The output of the timer is connected to the decade counter that counts the pulses. Before the Raspberry Pi shuts down, it triggers the second latching circuit to ON state initiating the countdown. At the tenth pulse, the decade counter triggers the main latching circuit to OFF state switching the entire circuit OFF. This gives the Raspberry Pi about 40 s to shut down safely before power is cut off. Fig. 4 shows the timer circuit.

### Power circuit

**Fig. 5:**
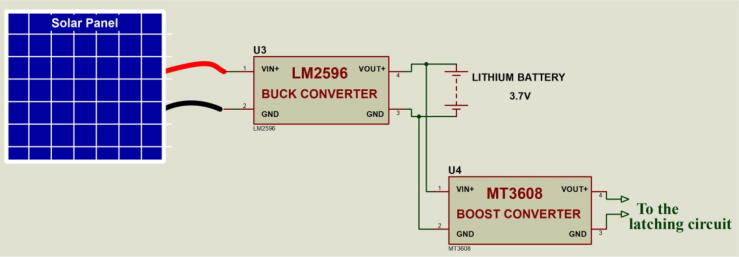
Power circuit.

This section comprises a solar panel, a buck converter and a boost converter. The solar panel charges the battery through the buck converter. The buck converter steps down the voltage of the solar from 18 V which is the rated voltage of the solar panel used to 4.2 V which is the rated charging voltage of the battery. Any solar panel that is rated between 5 V and 35 V may be used. The battery then supplies power to the system through the boost converter. The boost converter steps the battery voltage up from 3.7 V to 5.1 V which is the rated voltage of the Raspberry Pi. Fig. 5 shows the power circuit of the system.

### PCB development

The sections above were combined to create the schematic of the system provided in the ‘Hardware’ folder on KiCad software. The schematic was then converted to a PCB design. We used chemical etching to produce the PCB so the following instructions are for making the board using chemical etching. First, print the PCB design on glossy paper with toner. Transfer the printout to a copper cladded board by placing it face down over the board and press it with a hot iron box. Once the transfer is complete, remove the paper carefully and place the board in a solution of ferric chloride. Wear protective gears when working with ferric chloride. A REDOX reaction dissolves the unwanted copper leaving behind the copper covered by the toner which forms the tracks for the PCB.

**Fig. 6:**
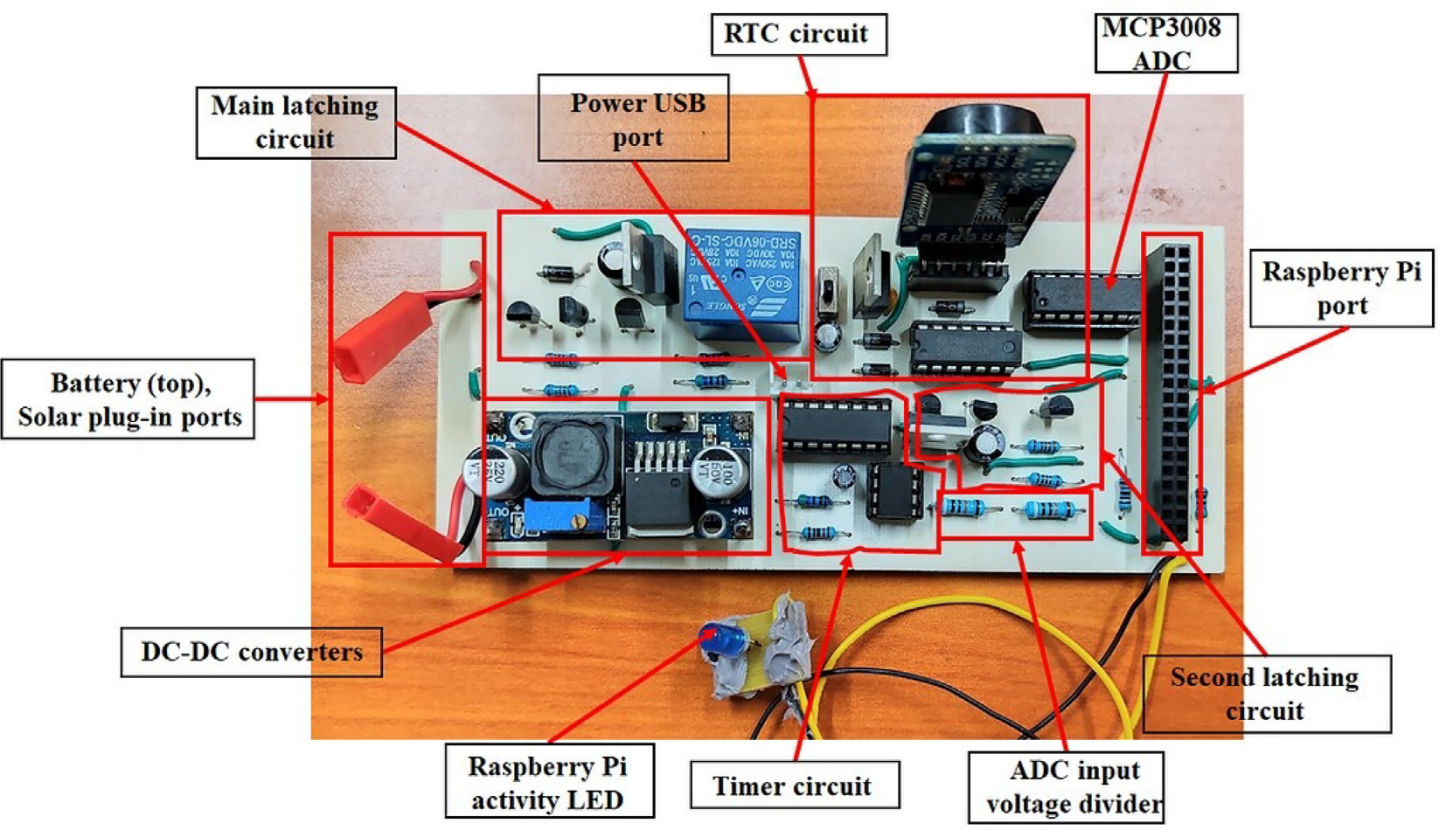
The main sections of the power supply board.

Once the PCB has been formed, rinse the board using water. Wipe off the toner using acetone. Proceed to drill holes on the designated points for components placement. Next, place the components into their respective points while observing their polarity where necessary and solder them. Prepare the battery and the solar panel by adding connectors with JST connector in order to make it possible to connect to the board. Fig. 6 shows the different sections of the power supply board.

## Operation instructions

**Fig. 7:**
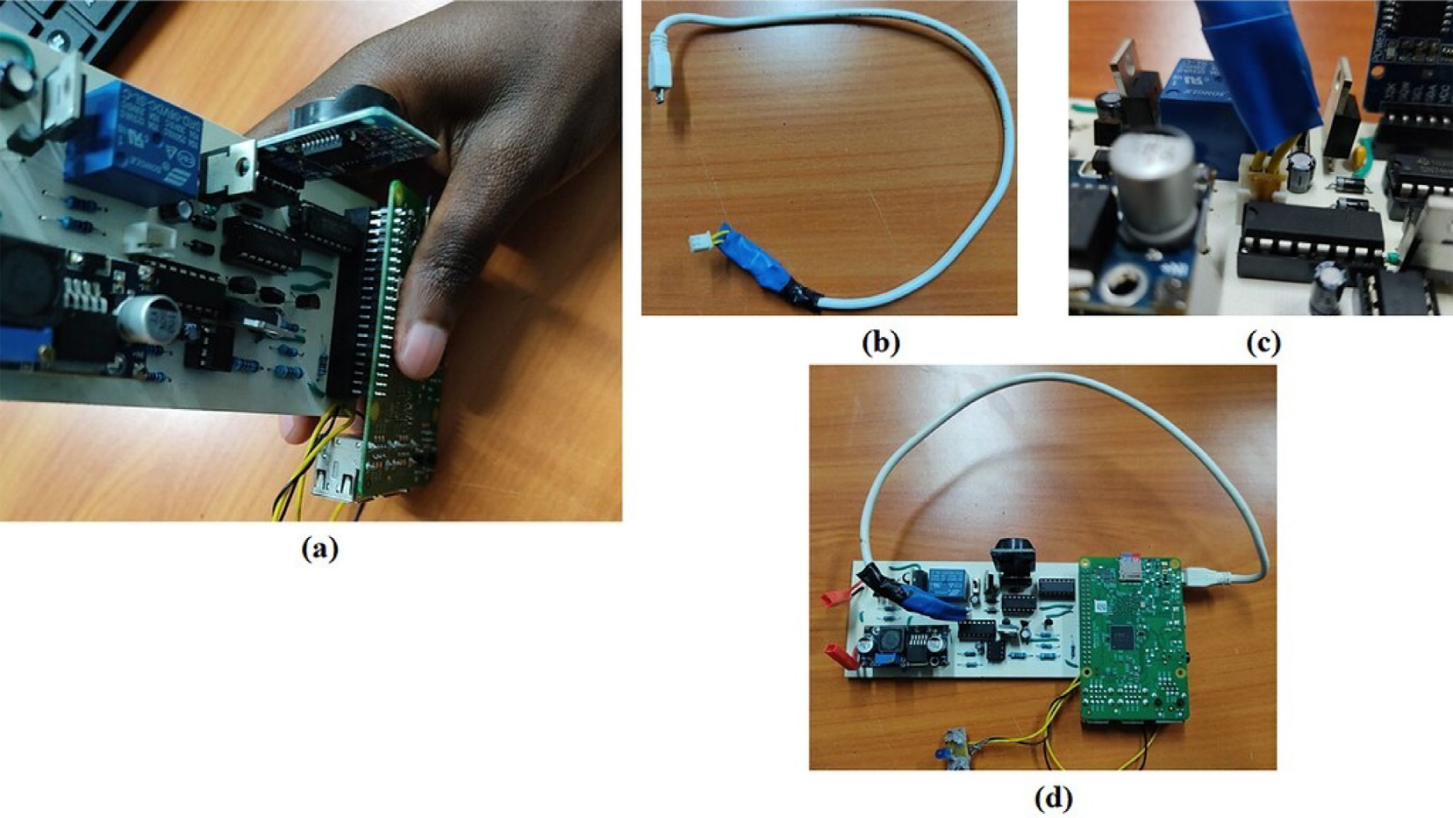
(a) Plugging the Raspberry Pi to the board, (b) a modified USB cable, (c) USB plugged to the power board and (d) USB plugged to the Raspberry Pi’s power port.

Before we use the board to power the Raspberry Pi, we need to install some requirements in the Raspberry Pi. Follow the instructions outlined in this repository (https://github.com/DeKUT-DSAIL/powering-raspberrypi/blob/main/README.md) to prepare the Raspberry Pi. Once the setup is complete, plug in the Raspberry Pi on the board and plug in the power USB as shown in Fig. 7.

**Fig. 8:**
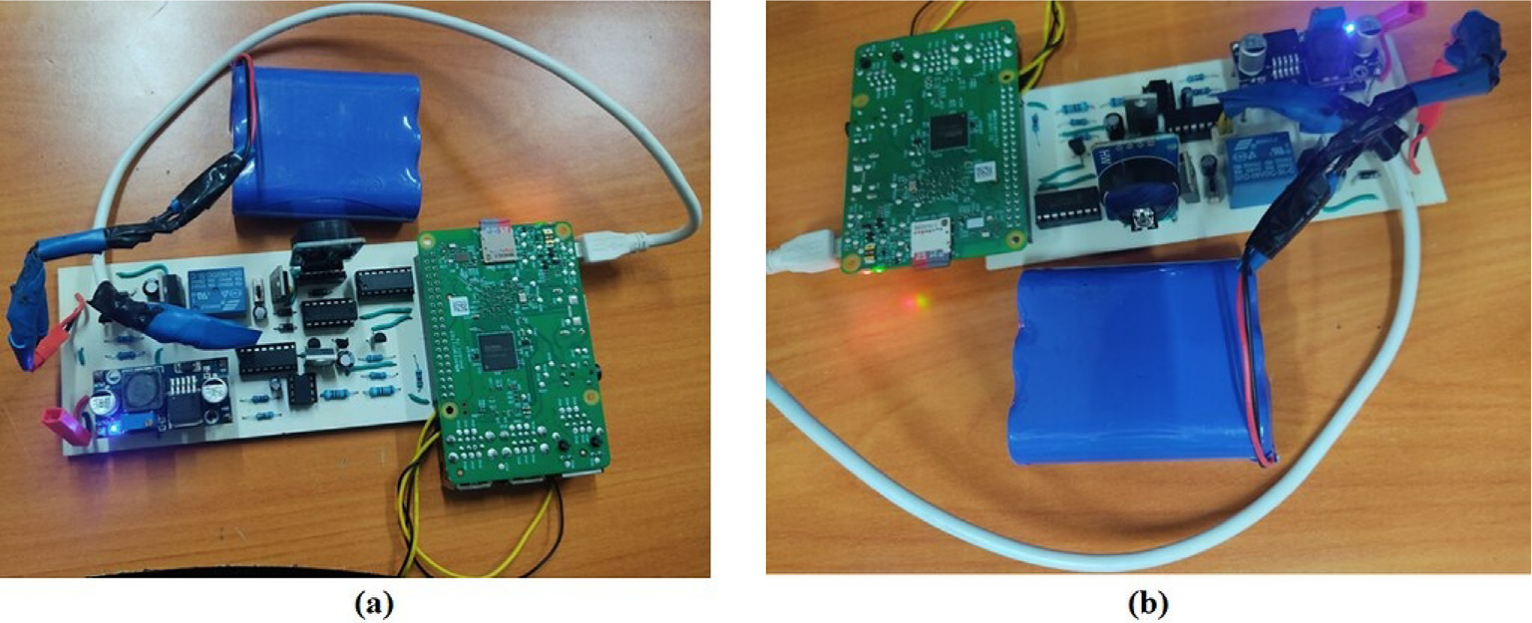
(a) Battery plugged into the board and (b)the system switched ON.

Next, plug in the battery to its JST port. Before plugging in the battery ensure the slide switch is OFF. Switch ON the system by sliding the SPDT slide switch and the system should turn ON as shown in Fig. 8.

**Fig. 9:**
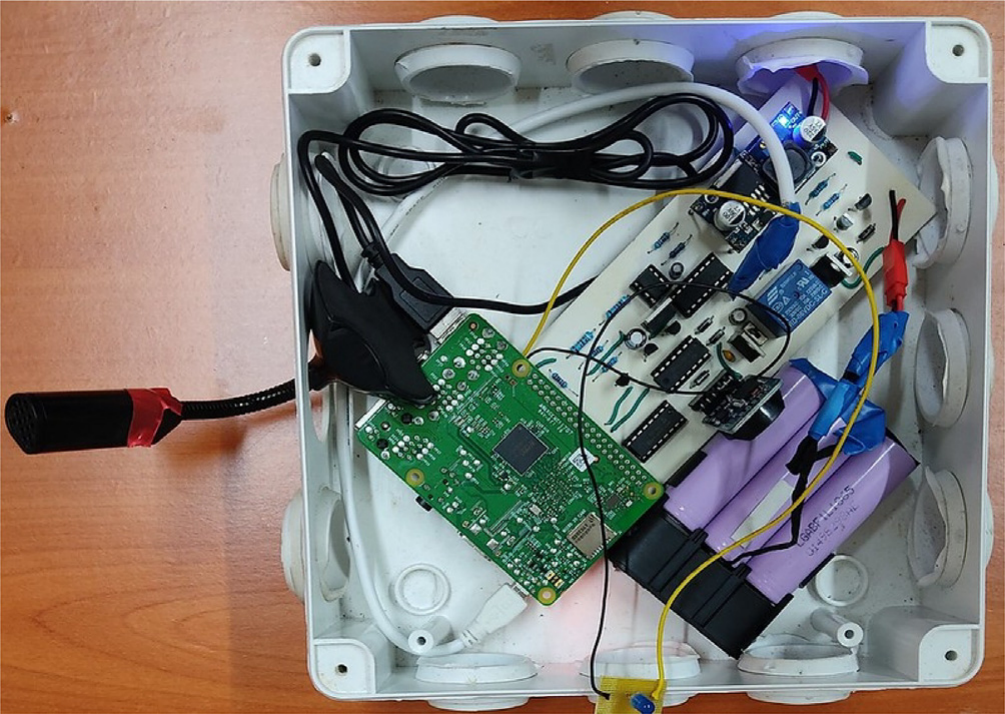
System placed in an adapter box for acoustic data collection.

For deployment, plug in the solar panel to its JST port and place the system in an enclosure to protect it. Fig. 9 shows the system setup for acoustic data collection.

## Validation and characterization

We have deployed our system for various ecological data collection tasks. We have used it to collect birds’ acoustic data, and camera trap images data at the Dedan Kimathi University of Technology Wildlife Conservancy. The system has proved to be very efficient in data collection in the field due to its autonomy.

### Birds’ acoustic data collection

Ecosystems contain vast amounts of acoustic data that can be used to tell the changes taking place in them [8]. Birds, especially, vocalise a lot and we can monitor our ecosystems by recording and analysing their sounds. To achieve this, we need to deploy acoustic sensors in the wild to collect data. An ideal acoustic sensor should record and process the data for us, reducing the need to deal with the large volumes of data that will be collected during its deployment. We have developed the DSAIL Bioacoustics System for acoustic data collection and processing. The sensor is based on the Raspberry Pi and is powered using the DSAIL Power Management Board [9].

We have deployed the acoustic sensor at the Dedan Kimathi University of Technology Wildlife Conservancy for data collection. Using the power supply board, we can control the time of the day we want the system to start recording birds. This makes it possible to have the system running when birds are most active. We have set the system to record birds from 5.00 am to 11.00 am when birds are most active and then it shuts down the rest of the day.

**Fig. 10:**
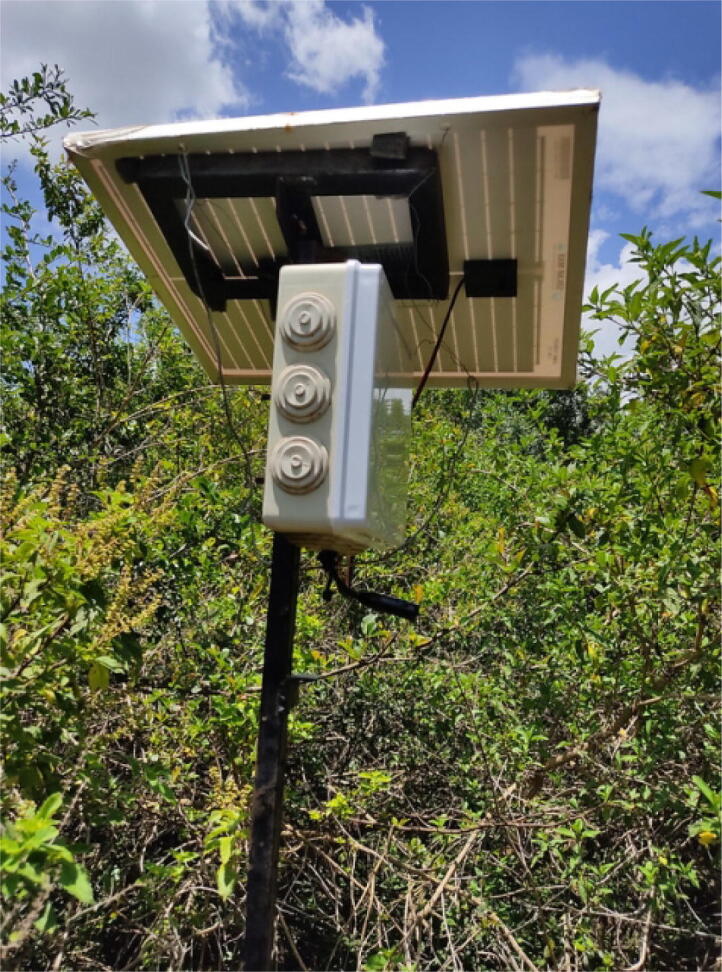
Acoustic system deployed for data collection at the University conservancy.

We have deployed the system for more than one year and it has collected over 500-hours long recordings. The recordings contain very diverse sounds of birds and non-birds sources. Various species of birds like the Hartlaub’s Turaco, Grey-backed Camaroptera and Tropical Boubou have been captured by the system. This data has the potential to develop the field of automatic acoustic classification of birds. We plan to use this data to develop machine learning algorithms to classify birds and load them in the Raspberry Pi. We will then deploy the system in our ecosystems and monitor them remotely. Fig. 10 shows the deployment of the system at the University’s Conservancy.

### A Raspberry Pi based camera trap

**Fig. 11:**
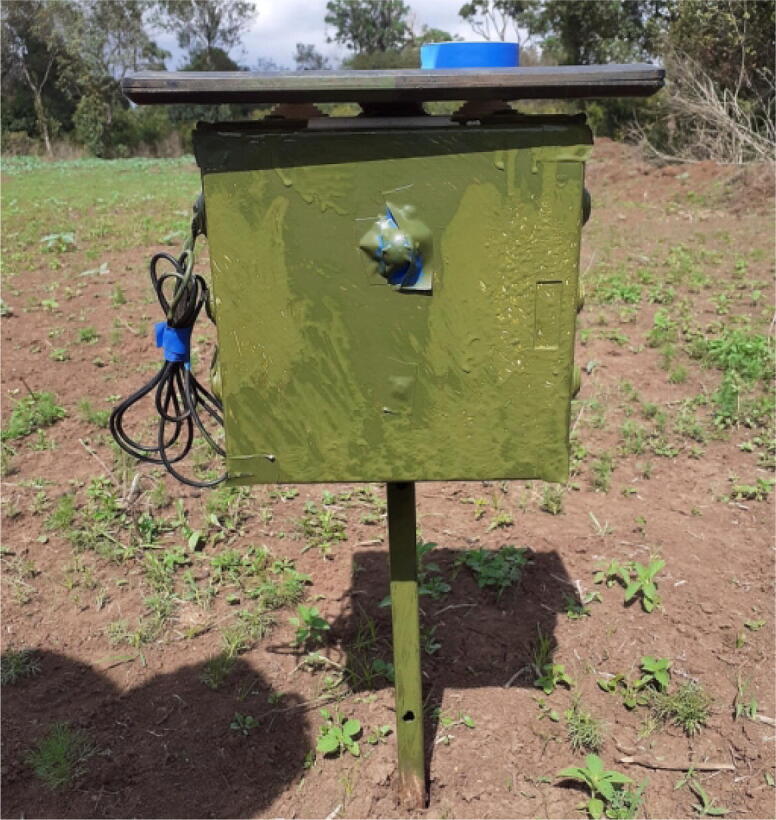
Camera trap deployment at the University Conservancy.

Other than acoustic sensors, camera traps are another form of noninvasive method of monitoring wildlife. Camera traps are deployed to capture images in the wild and using these images we can infer a lot about what is happening in our ecosystems [10]. We have developed 3 Raspberry Pi based camera traps and deployed them at the University Conservancy for image data collection. The camera traps are powered using the DSAIL Power Management Boards. The systems wake up at 6.00 am to 11.00 am then shut down and wake up again at 2.00 pm to 7.00 pm. We have managed to collect over 5000 images of animals. From this data, we can source a lot of information about the status of the wildlife in the conservancy [11], [12]. Fig. 11 shows the deployment of one of the camera traps.

### Power analysis

During a deployment, the Raspberry Pi periodically saves the battery voltage reading. Using these readings, it is possible to do a power analysis of the system. For the acoustic monitoring deployment, the system was set to operate from 5.00 am to 11.00 am when birds are most active. The system then shuts down the rest time of the day during which the battery gets charged. The system wakes up the following day and the cycle continues. This way we are able to operate the system optimally.

**Fig. 12:**
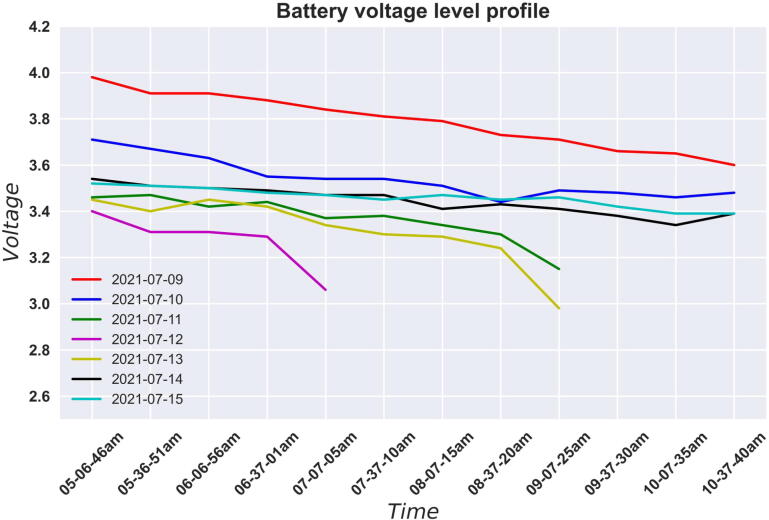
Plots of the battery voltage profile for different days between 5.00 am and 11.00 am.

If the battery voltage happens to drop to or below the cut-off voltage of the battery during the window of operation, the system will shut down and schedule for wake up the following day at 5.00 am. The battery will have been charged then if there was ample sunshine. If the battery happens to not have been charged enough to run the system, the system will shut down and schedule for wake up for the following day. This cycle will continue until the batter is charged enough to run the system. Fig. 12 shows a plot of the voltage profile of the battery during a deployment for the period 09/07/2021to15/07/2021:

From Fig. 12, we can observe the discharge profile of the battery during this deployment. The system worked throughout the scheduled window for four days out of the seven. For the other three days, the battery voltage dropped below the cut-off voltage of the battery set at 3 V and the system shut down. The three days are on 11th, 12th and 13th.

It is important to note that the system recovered on the 14th and 15th and it was able to operate during the whole window from 5.00 am to 11.00 am. We can also observe that the battery voltage reading at the beginning of most days is always higher than the last reading of the previous day. This means that the battery gets charged after the system has shut down.

## Conclusion

In this paper we have presented the DSAIL Power Management Board which is a power supply board that has been designed to power the Raspberry Pi autonomously using batteries and a solar panel allowing the use of the Raspberry Pi in off grid long term deployments. Our power management board enables the Raspberry Pi to work optimally on batteries by offering the following features: (1) ability to set the time the system will operate during the day and shut it down when not in use; (2) equips the Raspberry Pi with the ability to monitor the battery; (3) enables the Raspberry to shut down when the batteries get drained and wake up when the batteries get charged; (4) the RTC is used to set the time on the Raspberry Pi. These features make the DSAIL Power Management Board suitable for powering the Raspberry Pi during off-grid deployments. This paper has described in detail the design and fabrication of the system and verified the system's performance in two test data collection deployments.

## CRediT authorship contribution statement

**Gabriel Kiarie:** Conceptualization, Software, Writing – original draft. **Jason Kabi:** Validation. **Ciira wa Maina:** Resources, Supervision, Writing – review & editing.

## Declaration of Competing Interest

The authors declare the following financial interests/personal relationships which may be considered as potential competing interests: Ciira Maina reports financial support was provided by National Research Fund (NRF) Kenya. Ciira Maina reports financial support was provided by Google Inc. Ciira Maina reports financial support was provided by Canada’s International Development Research Centre (IDRC). Ciira Maina reports financial support was provided by Swedish International Development Cooperation Agency (Sida).
